# Opioid-reduced anesthesia based on esketamine in gynecological day surgery: a randomized double-blind controlled study

**DOI:** 10.1186/s12871-022-01889-x

**Published:** 2022-11-16

**Authors:** Teng Zhu, Xiaoyong Zhao, Meiyan Sun, Yan An, Wenwen Kong, Fanceng Ji, Guizhi Wang

**Affiliations:** 1 School of Anesthesiology, WeiFang Medical University, WeiFang, 261053 China; 2 Department of Anesthesiology, WeiFang, People’ Hospital, WeiFang, 261000 China

**Keywords:** Esketamine, Opioid-reduced anesthesia, PONV, Hemodynamics

## Abstract

**Background:**

Opioid-reduced anesthesia may accelerate postoperative rehabilitation by reducing opioid-related side effects. The objective was to investigate the feasibility of opioid-reduced general anesthesia based on esketamine and to observe postoperative nausea and vomiting (PONV), postoperative pain, hemodynamics and other adverse reactions in gynecological day surgery compared with the traditional opioid-based anesthesia program.

**Method:**

This study was conducted as a prospective parallel-group randomized controlled trial. A total of 141 adult women undergoing gynecological day surgery were included. Patients were randomly assigned to receive traditional opioid-based anesthesia (Group C) with alfentanil, or opioid-reduced anesthesia (a moderate-opioid group (Group MO) and low-opioid group (Group LO) with esketamine and alfentanil). For anesthesia induction, the three groups received 20, 20, 10 μg/kg alfentanil respectively and Group LO received an additional 0.2 mg/kg esketamine. For maintenance of anesthesia, the patients in Group C received 40 μg/kg/h alfentanil, and those in Group MO and Group LO received 0.5 mg/kg/h esketamine.

**Results:**

Patients in the three groups had comparable clinical and surgical data. A total of 33.3% of patients in Group C, 18.4% of patients in Group MO and 43.2% of patients in Group LO met the primary endpoint (*p* = 0.033), and the incidence of nausea within 24 hours after surgery in Group MO was lower than in Group LO (*p* < 0.05). The extubation time, median length of stay in the hospital after surgery and visual analog scale (VAS) of postoperative pain were equivalent in the three groups. The frequencies of adverse hemodynamic events in the MO 1(0, 2) and LO 0(0, 1) groups were significantly decreased (*p* < 0.05). Compared with Group C, the median length of stay in the postanesthesia care unit (PACU) in Group LO was increased, 60.0 (36.25, 88.75) vs. 42.5 (25, 73.75) minutes (*p* < 0.05).

**Conclusions:**

Opioid-reduced anesthesia based on esketamine is feasible and provides effective analgesia for patients. Esketamine provided a positive analgesic effect and the opioid-reduced groups showed more stable hemodynamics. However, less or no use of opioids did not result in a more comfortable prognosis.

**Trial registration:**

This study was registered at Chictr.org.cn (NO. ChiCTR2100053153); November 13, 2021.

## Background

Over the past 2000 years, opioids and their derivatives have become the cornerstone of the treatment of moderate to severe pain. Opioids are often used for intraoperative and postoperative pain management during the perioperative period [1]. However, perioperative opioid use has also been associated with significant adverse side effects [2], including nausea, gastrointestinal paralysis, delirium and hypoxemia. One study in 2013 reported that 12% of surgeries had opioid-related adverse events [3]. As an alternative, many nonopioid analgesics are currently available, including acetaminophen, nonsteroidal anti-inflammatory drugs, alpha-2 agonists, N-methyl-d-aspartate (NMDA) receptor antagonists, gabapentins and antidepressants [4].

The efficacy of perioperative intravenous ketamine as an adjunctive analgesic has been well established. Low-dose (< 1 mg/kg) ketamine inhibited NMDA receptors in nociceptive neurons and activated the downward pain inhibition pathway [5]. Combined with opioids, ketamine can enhance the effect of analgesia and reduce opioid consumption, as well as reduce perioperative pain-related neuroticism which would cause postoperative pain [6]. Esketamine is a dextroisomer of ketamine, and a chiral cyclohexanone with a strong analgesic effect. Additionally, esketamine can be used in combination with sedative hypnotics for induction and general anesthesia, reducing propofol consumption by 20% [7], or as a supplement to local anesthesia. At present, some studies [8–11] have shown that ketamine and esketamine can be combined with other drugs to implement opioid-free anesthesia.

A meta-analysis and systematic review found that PONV was significantly reduced with opioid-free anesthesia [1]. As one of the independent risk factors for PONV [12], female sex is significantly increases the probability of PONV. On this account, we conducted a randomized controlled trial in gynecological day surgery to investigate whether opioid-reduced anesthesia based on esketamine reduces opioid related adverse reactions and accelerates the rehabilitation of patients.

## Methods

### Study design and ethics

This study was designed to be a double-blind parallel randomized controlled trial and applied the opioid-reduced anesthesia program to gynecological day surgery to observe the effects on patients’ hemodynamics, PONV, postoperative pain and other adverse reactions. Ethics approval was obtained from the Ethics Committee of the Weifang People’s Hospital (2021-030) on November 11, 2021. The study was registered in Chinese Clinical Trial Registry (November 13, 2021; Chinese Clinical Trial registry, No. ChiCTR2100053153). Written informed consent was obtained from all participants. The trial report complies with the Consolidated Standards of Reporting Trials (CONSORT) checklist.

### Participants

Eligible patients were adult women scheduled for hysteroscopy and cervical conization day surgery at Weifang People’s Hospital. The exclusion criteria were: 1) morbid obesity, body mass index (BMI) > 30 kg/m^2^; 2) coronary artery disease, liver and kidney dysfunction, and neuromuscular and psychiatric disorders; 3) a history of cerebral infarction, myocardial infarction, and severe arrhythmia; 4) a history of chronic pain or use of any sedatives or analgesics; and 5) allergies to any drug involved in the study. All patients enrolled in this study were evaluated by anesthesiologists above the attending doctor before anesthesia and signed informed consent forms. If the patient refused to participate in the study or the surgical method was changed, the study was stopped. The study protocol had no important harmful or unintended effects on participants.

### Outcomes

The primary endpoint was the incidence of postoperative nausea within 24 h after surgery. The secondary endpoints included the incidence of postoperative vomiting, pain scores evaluated by the VAS, length of stay in the PACU until discharge criteria according to the Aldrete’s modified postanesthetic recovery score [13], adverse hemodynamic events during the perioperative period, postoperative length of hospital stay and other adverse reactions.

### Randomization and blinding

Patients were randomly allocated to the two interventions or the control group. The computer-generated random allocation sequence was randomly created by an independent investigator using Excel 2016 with a 1:1:1 allocation randomly. Participants and outcome evaluators were blinded to group assignments. Because of the significant differences between anesthetic techniques, the anesthesia providers could not be blinded.

### Procedures

The tow opioid-reduced groups were based on previously reported approaches [14–17] and the instructions and were assessed for feasibility in a pilot series preceding this study.

After verifying the patient’s information, routine monitoring was established, and baseline values were obtained. Then, venous access was obtained, and a crystalloid solution was started. All patients received flurbiprofen axetil 50 mg to relieve inflammatory pain and then dexamethasone 5 mg for PONV prophylaxis based on preoperative risk stratification with Apfel’s simplified PONV risk score [18].

All patients received preoxygenation after verification. For anesthesia induction, all patients received propofol at a loading dose of 2 mg/kg intravenous injection in one minute. Patients in Group C and Group MO received 20 μg/kg alfentanil, and patients in Group LO received alfentanil 10 μg/kg mixed with 0.2 mg/kg esketamine. Then, the patients in the three groups received 0.2 mg/kg mivacurium chloride, followed by laryngeal mask insertion for three minutes. The ventilator settings were adjusted to maintain normoxia (SpO_2_ > 97%) and normocapnia (35 < PETCO_2_ < 45 mmHg). Body temperature management was initiated to maintain normothermia. For maintenance of anesthesia, all patients in there groups received 0.5 mg/kg/h propofol. The patients in Group C received 40 μg/kg/h alfentanil, and those in Group MO and Group LO received 0.5 mg/kg/h esketamine. Bradycardia was defined as a heart rate (HR) below 50 beats per minute [19] and hypotension was defined as a mean arterial pressure (MAP) below 65 mmHg [20]. Hypotension or bradycardia occurring at each intraoperative time point was recorded as a hemodynamic adverse event, and summarized after operation as the frequency of adverse events in the perioperative period. Patients with bradycardia and hypotension were given 0.5 mg atropine or 0.6 mg ephedrine by intravenous injection. Considering the specific pharmacokinetics of the drugs used in each arm of the trial, all anesthetic agents were stopped after the end of surgery.

After tracheal extubation, patients were transferred to the PACU and assessed for nausea, vomiting, pain and other adverse reactions. For the remedial treatment of postoperative pain, we recommended oral ibuprofen (0.4 g, bid) for three days after discharge. For antiemetic rescue treatment, patients received drinking water, chewing gum or 5 mg tropisetron intravenous injection. Postoperative pain and antiemetic therapy were standardized according to institutional regulations. Patients were assessed for discharge readiness in accordance with the criteria of Aldrete’s Modified Postanesthetic Recovery Score, and the length of stay in the PACU was defined from the time of admission until these criteria were met.

### Outcome assessments and data collection

There were nine time points in the whole operation, including the patient entering the operating room (T0), before anesthesia induction (T1), after anesthesia induction but before laryngeal mask insertion (T2), laryngeal mask fixation (T3), the beginning of the surgery (T4), 5 minutes into surgery (T5), 10 minutes into surgery (T6), 15 minutes into surgery (T7),stopping of the anesthesia (T8) and 1 minute after extubation (T9). The MAP and HR of patients at each time point were recorded. If the MAP or HR reached the standard we set, an adverse hemodynamic event was considered to have occurred. The total frequence of hemodynamic adverse events during the operation was recorded. We also recorded the drug dosage, total anesthesia time, extubation time, length of stay in the PACU and in the hospital, postoperative pain and other perioperative adverse reactions (vertigo, excessive oral secretion, etc.) of all patients. Excessive oral secretion was defined as excessive secretion that patients could not remove by themselves, and that needed to be removed by an aspirator after extubation. Patients were followed up at two postoperative time points (within the PACU and on the first day of discharge) to evaluate the incidence of PONV.

### Statistical analysis

According to the pretest results, we calculated the sample size with PASS 15.0 software using the incidence of nausea within 24 hours as the primary study endpoint. Sample size calculations resulted in *n* = 41 patients per group to achieve a power of 90% with a type 1 error of 0.05 to reject the primary null hypothesis that there would be no difference in the primary outcome between all three treatment arms. With an estimated sample loss rate of 15% estimated, we finally decided to include 150 patients.

Data are summarized as the mean (standard deviation), median (interquartile range [IQR]), or number (%). Categorical data were analyzed with Fisher’s exact test or the X^2^ test. One-way ANOVA was used for normally distributed data assessed by the Shapiro–Wilk test. The least-significant difference test (LSD) was used for post hoc testing. Continuous variables that did not conform to a normal distribution were assessed with the Kruskal-Wallis test. Bonferroni correction was performed in paired comparisons. Two-sided *p* values < 0.05 were considered significant. All statistical analyses were performed by SPSS Statistics 25.0. The statistical analysis plan was approved by the authors before the analyses began.

## Results

### Patient characteristics

**Fig. 1:**
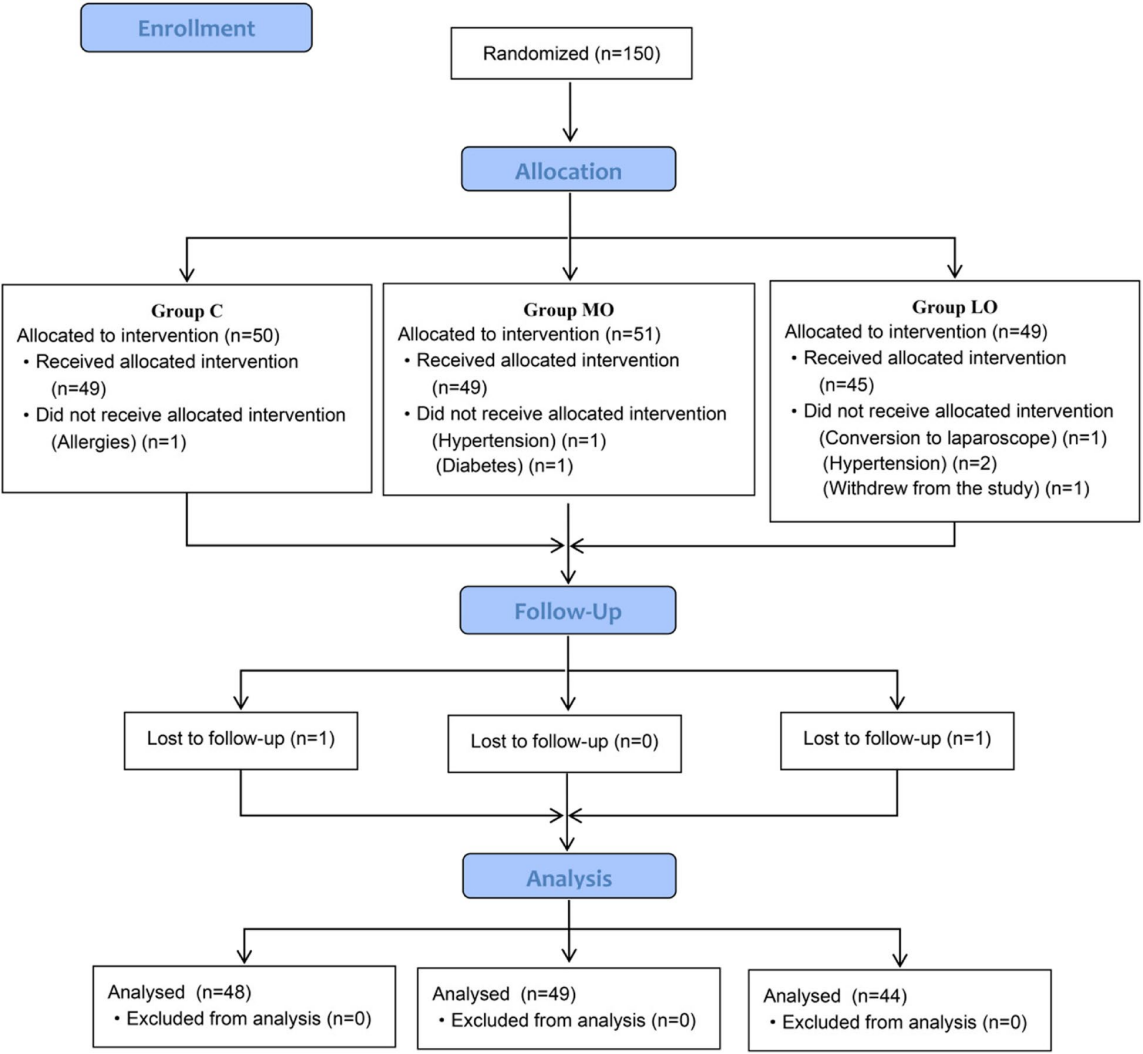
Patients enrollment diagram

A total of 150 patients were enrolled in the study, of whom 141 patients were available for the primary analysis. Five patients were excluded because of hypotension, diabetes and allergies. Two patients were excluded because of conversion to laparoscopy or withdrawal from the study. And two patients were lost to follow-up (Fig. 1).

Clinical characteristics were comparable in both groups (Table 1).

**Table 1:** Baseline patient characteristics and perioperative data for all patient and by group allocation, data are summarized by number (%), median (interquartile range) or mean (standard deviation)

	All patients (*n* = 141)	Group C (*n* = 48)	Group MO (*n* = 49)	Group LO (*n* = 44)	*p*-Value
Age	41.89 ± 9.29	41.58 ± 7.41	41.49 ± 9.85	42.68 ± 10.58	0.796
Weight (kg)	61.08 ± 7.71	60.71 ± 6.67	60.81 ± 8.76	61.8 ± 7.66	0.762
BMI (kg/m^2^)	23.37 ± 2.96	23.09 ± 2.92	22.98 ± 2.94	24.1 ± 2.95	0.139
ASA score					0.221
I	66 (46.8%)	27 (56.3%)	19 (38.8%)	20 (45.5%)	
II	75 (53.2%)	21 (43.8%)	30 (61.2%)	24 (54.5%)	
PONV risk score					0.956
2	84 (59.6%)	28 (58.3%)	30 (61.2%)	26 (59.1%)	
3	57 (40.4%)	20 (41.7%)	19 (38.8%)	18 (40.9%)	
Surgical procedure					0.497
Hysteroscopy	107 (75.9%)	36 (75%)	35 (71.4%)	36 (81.8%)	
Cervical conization	34 (24.1%)	12 (25%)	14 (28.6%)	8 (18.2%)	
Anesthesia Time (min)	20 (14, 27.5)	20 (14, 34.5)	18 (13, 25.5)	21 (15, 29.5)	0.222
Extubation time (min)	5 (4, 6)	5 (3, 6)	5 (4, 6)	5 (3, 6)	0.692
Length of postoperative hospital stay (h)	3 (2, 4)	3.5 (2, 4)	3 (2, 4)	2.5 (2, 3.5)	0.055
Length of stay in the PACU (min)	50 (30, 80)	42.5 (25, 73.75)	50 (30, 105)	60 (36.25, 88.75)*	0.036
Alfentanil consumption (ug/kg)	20 (10, 25.6)	30.3 (25.2, 37.8)	20 (18.69, 20)a	10 (9.75, 10)*	0.000

^*^Compared with Group C, *p* < 0.05

Apfel’s PONV risk score did not differ significantly among the three groups (*p* = 0.956); approximately 59.6% of all patients had a risk of 39% to develop PONV and other patients had a risk of 61%. Hysteroscopy and cervical conization surgery were the main operations in gynecological day surgery. The type of surgery and overall duration of anesthesia were comparable between groups, and the incidence of PONV did not differ by surgical procedure (*p* = 0.497). The median intraoperative alfentanil consumption was 30.26 (25. 19, 35.92) μg/kg in Group C, 20 (18. 69, 20) μg/kg in Group MO and 10 (9. 75, 10) μg/kg in Group LO (*p* < 0.05) (Table 1).

### Study endpoints

#### Postoperative nausea and vomiting

A total of 33.3% of patients in Group C, 18.4% of patients in Group MO and 43.2% of patients in Group LO met the primary endpoint (*p* = 0.033), and the incidence of nausea within 24 hours after surgery in Group MO was significantly decreased compared with that in Group LO (*p* < 0.05). A total of 8.3% of patients in Group C, 2% of patients in Group MO and 20.5% of patients in Group LO suffered from vomiting within 24 hours after operation (*p* = 0.013), and the incidence was lower in Group MO than in Group LO (*p* < 0.05). Compared with Group LO, the incidence of nausea in the PACU and vomiting on the first day after the operation in Group MO were significantly decreased (*p* < 0.05). The incidence of nausea on the first day after the operation was lower in Group MO (*p* < 0.05) (Table 2).

**Table 2:** Incidence of postoperative nausea and vomiting in PACU and the first day after operation

	All patients (*n* = 141)	Group C (*n* = 48)	Group MO (*n* = 49)	Group LO (*n* = 44)	*p*-Value
Nausea within 24 h	44 (31.2%)	16 (33.3%)	9 (18.4%)#	19 (43.2%)	0.033
Vomiting within 24 h	14 (9.9%)	4 (8.3%)	1 (2%)#	9 (20.5%)	0.013
PACU					
Nausea	16 (11.3%)	4 (8.3%)	2 (4.1%)#	10 (22.7%)	0.013
Vomiting	11 (7.8%)	2 (4.2%)	2 (4.1%)	7 (15.9%)	0.066
The first day after operation					
Nausea	39 (27.7%)	15 (31.3%)	7 (14.3%)#*	17 (38.6%)	0.025
Vomiting	10 (7.1%)	3 (6.3%)	0 (0%)#	7 (15.9%)	0.006

* Compared with Group C, *p* < 0.05

# Compared with Group LO, *p* < 0.05

#### Recovery, postoperative pain and perioperative hemodynamics

**Fig. 2:**
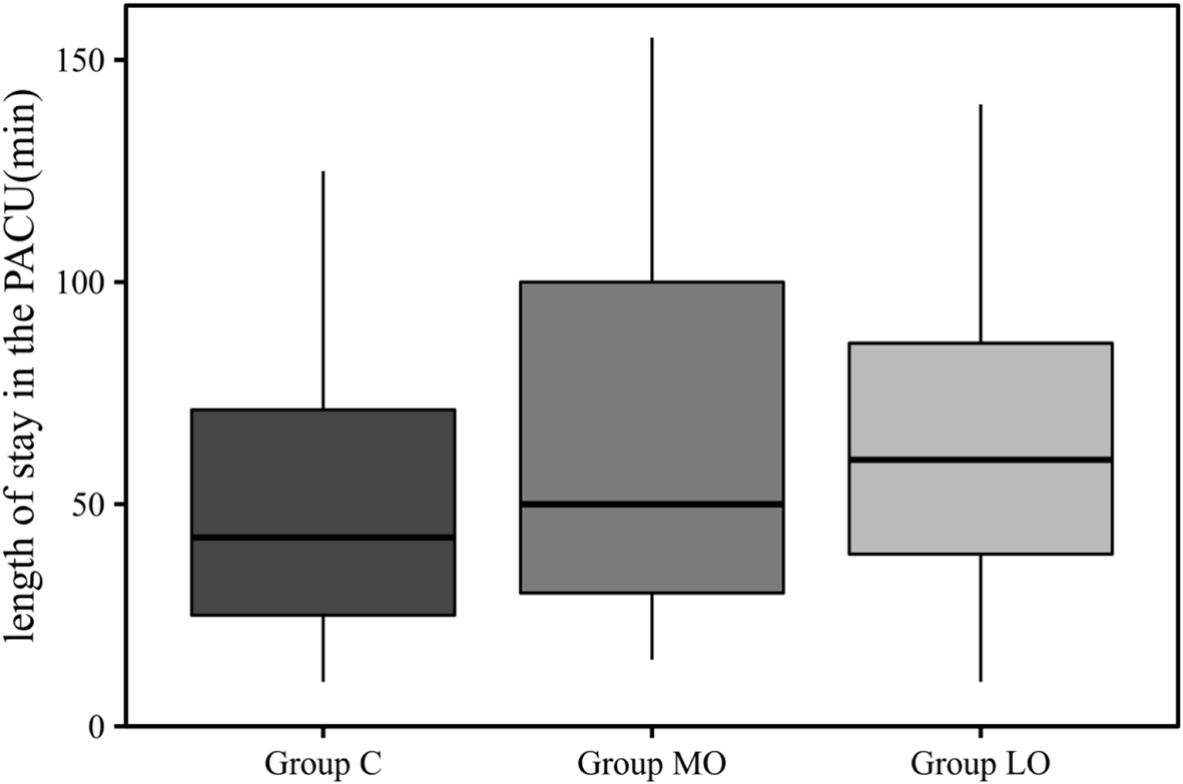
The length of stay in the PACU

The length of stay in the PACU was increased in Group LO compared with Group C, with a median length of 60(36. 25, 88.75) vs. 42.5 (25, 73.75) minutes (*p* < 0.05) (Table 1) (Fig. 2).

**Fig. 3:**
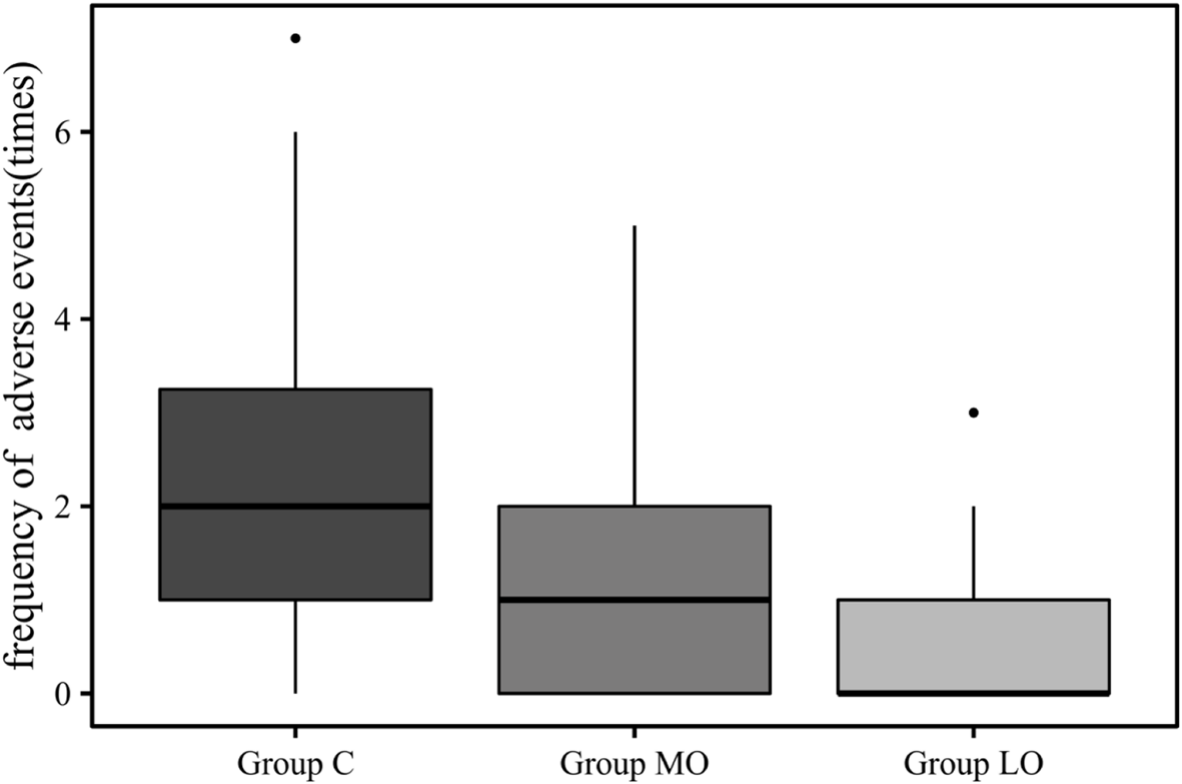
The frequency of periopertive hemodynamic adverse event

The VAS scores of postoperative pain were not significantly different among the three groups. Only one patient in Group LO asked for the extra remedial treatment of postoperative pain. Compared with Group C, the number of patients in Group LO with bradycardia (12/25% vs. 2/4.5%, p < 0.05) and hypotension (35/72.9% vs. 20/45.5%) was significantly decreased. (Table 3). The median frequency of hemodynamic adverse events in Group C (2 (1, 3.75)) was higher than that in Group MO (1 (0, 2))and Group LO (0 (0, 1)) (*p* < 0.001) (Table 3) (Fig. 3).

**Table 3:** Incidence and frequency of perioperative hemodynamic adverse event

	All patient (*n* = 141)	Group C (*n* = 48)	Group MO (*n* = 49)	Group LO (*n *= 44)	*p*-Value
Hemodynamics					
Bradycardia	21 (14.9%)	12 (25%)	7 (14.3%)	2 (4.5%)*	0.022
Hypotension	81 (57.4%)	35 (72.9%)	26 (53.1%)	20 (45.5%)*	0.022
Frequency of adverse events	1 (0, 2)	2 (1, 3.75)	1 (0, 2)*	0 (0, 1)*	0.000
Postoperative pain	0 (0, 2)	0 (0, 2)	0 (0, 2)	0 (0, 2)	0.929

*Compared with the Group C, *p* < 0.05

#### Perioperative adverse reactions

The number of patients who suffered vertigo in Group LO (9/20.5%)was significantly increased (1/2.1% in Group C and 1/2% in Group MO, *p* < 0.05). The same is true for excessive oral secretion. More patients in Group LO had increased salivary secretion that needed to be removed by an aspirator (0/0% in Group C, 4/8.2% in Group MO, and 17/38.6% in Group LO, *p* < 0.05). (Table 4). There was one patient in Group C suffered from hiccups, and none of the patients in any of the three groups developed headaches or choking.

**Table 4:** Perioperative adverse reactions

	All patient (*n* = 141)	Group C (*n* = 48)	Group MO (*n* = 49)	Group LO (*n* = 44)	*p*-Value
Postoperative Vertigo	11 (7.8%)	1 (2.1%)#	1 (2%)#	9 (20.5%)	0.001
Excessive oral secretion	21 (14.9%)	0 (0%)#	4 (8.2%)#	17 (38.6%)	0.000

# Compared with the Group LO, *p* < 0.05

## Discussion

The objective was to investigate the feasibility of opioid-reduced general anesthesia based on esketamine and to observe postoperative nausea and vomiting (PONV), postoperative pain, hemodynamics and other adverse reactions in gynecological day surgery compared with the traditional opioid-based anesthesia program. In this study, Group MO and Group LO showed more stable hemodynamics than the traditional opioid anesthesia group. There was no significant difference in postoperative pain scores among the three groups. Group LO resulted in significant prolongation of PACU retention and aggravated PONV.

Although laparoscopy and gynecological surgery have been identified as independent risk factors for PONV, the general demand for opioids increases the risk of PONV by approximately 80% in women undergoing these operations [21]. Apfel’s simplified PONV risk score was used to stratify the risk of PONV in the enrolled patients, to reduce the factors related to PONV to a great extent. There is evidence [11] that opioid-free anesthesia can reduce the incidence and severity of PONV. In contrast to our results, however, there was no significant difference in the incidence of PONV between Group C and Group MO, whether at the PACU or on the first day after operation. However, the incidence of PONV was significantly increased in Group LO. This showed that opioid-reduced anesthesia based on esketamine does not significantly reduce PONV, and less or no use of opioids makes PONV more serious. We think that the performance of PONV may be related to the side effect of esketamine [22]. In Group LO, the doses of alfentanil were further reduced, but the doses of esketamine were increased. An excessive dosage of esketamine made the patients need more time to overcome the discomfort. The PONV, vertigo and excessive oral secretion in Group LO led to an extension of the retention time at the PACU.

We found that, compared with Group C, the incidence of perioperative hypotension and bradycardia in Group LO was significantly decreased. In addition, the frequency of hemodynamic adverse events in the two opioid-reduced groups was lower than that in Group C. Esketamine has the characteristics of sympathetic nerve activation, which is comprehensively manifested as increased HR and blood pressure, improving perioperative hemodynamic stability [23]. Topcuoglu et al. [24] reported that 0.5 mg/kg ketamine combined with 2.5 mg/kg propofol improved intubation conditions through sympathomimetics. Propofol combined with esketamine has good safety and high reliability [16], obtaining more stable hemodynamics and reducing inflammatory and adverse reactions to promote the postoperative cognitive function recovery and rehabilitation of elderly patients.

Esketamine provided a good analgesic effect for gynecological day surgery in our clinic. Ketamine and esketamine are NMDA receptor antagonists that can inhibit inflammatory hyperalgesia. They may also enhance the anti-nociceptive effect induced by opioids. Compared with traditional ketamine, esketamine has a stronger analgesic effect and a higher clearance rate in vivo [25]. In the study of Edwards et al. [26], after 5 days of rat hind paw inflammation induced by Freund’s complete adjuvant, administration of ketamine and esketamine produced a slight but significant anti-nociceptive effect and reduced the edema of the hind paw after inflammation to a certain extent. Mark et al. [27] succeeded in reducing the prescription amount of opioids in patients who underwent abdominal surgery, while there was no significant difference in pain score or pain drug supplement request. This is consistent with our results. There was no significant difference in VAS score among the three groups and only one patient in Group LO asked for extra remedial treatment for postoperative pain. This confirmed the positive effect of esketamine on perioperative pain management.

### Limitations

To research the opioid-reducing effect of esketamine, we did not supplement with drugs other than those mentioned above. Others [8, 11] often advocate for the combined application of dexmedetomidine, lidocaine, esketamine and sevoflurane to completely replace opioids. During the preliminary experiment, we considered adding an opioid-free anesthesia group which may completely replace alfentanil with esketamine for anesthesia induction and maintenance. However, we found that high doses of esketamine may result in other side effects, such as dizziness, PONV, hypertension and salivary secretion. Considering the comfort of patients and medical ethics, we abandoned this plan and used Group LO as a substitute for the opioid-free group. In addition, we adopted the following methods to address PONV in the PACU: the provision of drinking water, chewing gum or antiemetic drugs. This may reduce the incidence of PONV at the second time point.

## Conclusion

Opioid-reduced anesthesia based on esketamine in patients undergoing gynecological day surgery is feasible and easy to perform. Esketamine provided a positive analgesic effect and the opioid-reduced groups showed more stable hemodynamics than the control group. Moreover, we found that a significant reduction in opioids in Group LO did not decrease the incidence of PONV and prolonged the rehabilitation of patients. Based on the limitations of our study design, it cannot be concluded that opioids can be completely replaced with esketamine alone. Opioid-free anesthesia requires the combined use of multiple drugs to provide comfortable anesthesia and better prognosis.

## Data Availability

The datasets used and analyzed during the current study are available from the corresponding author on reasonable request. Guizhi Wang e-mail: wgz@wfmc.edu.cn

## Abbreviations

PONV: Postoperative nausea and vomiting
PACU: Post anesthesia care unit
HR: Heart rate
MAP: Mean arterial pressure
BMI: Body mass index
VAS: Visual analogue scale
LSD: Least-significant difference test
